# Evaluation of the Effect of Thrombolytic Therapy on Inflammatory Markers in Patients With Acute Pulmonary Embolism

**DOI:** 10.7759/cureus.74926

**Published:** 2024-12-01

**Authors:** Muhammed Fatih Kaleli, Ahmet T Sahin, Yakup Alsancak

**Affiliations:** 1 Cardiology, Faculty of Medicine, Necmettin Erbakan University, Konya, TUR; 2 Cardiology, Beyhekim Training and Research Hospital, Konya, TUR

**Keywords:** acute pulmonary embolism, inflammatory mediators, intravenous thrombolytic therapy, multiple inflammation index, systemic immune inflammation index

## Abstract

Background: Acute pulmonary embolism (APE) is a serious cardiovascular condition characterized by high rates of morbidity and mortality, with inflammation playing a significant role in the severity of the disease and patient outcomes. This study aimed to evaluate the effects of thrombolytic therapy on inflammatory markers in patients diagnosed with APE.

Study design and methods: The study was conducted retrospectively on 138 individuals, 69 with pulmonary embolism and 69 without pulmonary embolism (control), who were admitted to Necmettin Erbakan University between January 2019 and April 2023. Demographic information, C-reactive protein (CRP), white blood cells (WBC), neutrophils, platelets, systemic immune-inflammation index (SII), neutrophil-lymphocyte ratio (NLR), platelet-to-lymphocyte ratio (PLR), multi-inflammatory index (MII-1 and MII-2), hemoglobin, and lymphocyte-to-C-reactive protein ratio (LCR) levels of the cases were evaluated. The inflammatory markers were measured before and after thrombolytic therapy.

Results: Levels of CRP, WBC, SII, NLR, and MII-1 were significantly increased in the pulmonary embolism group compared to the control group (p<0.05). In contrast, there was a notable decrease in hemoglobin, MII-2, and LCR levels (p<0.05). In the pulmonary embolism group, levels of CRP, WBC, neutrophils, lymphocytes, hemoglobin, SII, NLR, and MII-1 were significantly lower after thrombolytic treatment compared to their levels before the treatment (p<0.05). Conversely, the post-thrombolytic treatment group exhibited significantly higher levels of platelets, PLR, and LCR compared to the pre-thrombolytic treatment group (p<0.05).

Conclusions: The findings indicate that novel inflammatory markers, such as SII, NLR, and MII, are elevated in APE and tend to decrease following thrombolytic therapy. This suggests that these markers could be useful for monitoring treatment effectiveness and predicting patient outcomes. However, further research is necessary to confirm their clinical utility.

## Introduction

Acute pulmonary embolism (APE) is the third most common acute cardiovascular disease, following myocardial infarction and stroke [1]. It is frequently encountered in emergency departments, with an estimated annual incidence ranging from 39 to 115 cases per 100,000 people [2]. The risk of APE increases with age, and the mortality rate associated with it is quite high. The clinical signs and symptoms of APE, such as sudden onset of shortness of breath (dyspnea), chest pain, and episodes of fainting (syncope) or near-fainting (presyncope), are nonspecific. Therefore, rapid diagnosis of APE is crucial. Currently, the gold standard for diagnosing APE is computed tomography pulmonary angiography (CTPA) [3]. It is advisable to use a diagnostic algorithm that incorporates a clinical decision rule. This should include the patient’s medical history, physical examination findings, and D-dimer testing for individuals with clinically suspected APE. This approach aids in excluding the disease and minimizes the need for CTPA scans [4].

The mechanisms underlying APE are related to increased blood clotting, damage to the endothelial lining, and inflammation [5]. Multiple studies have confirmed the inflammatory response in APE, indicating that inflammatory markers may be valuable for diagnosing and prognosing these cases [6-8]. Systemic inflammation can be evaluated using various biochemical and hematological markers, such as the neutrophil-to-lymphocyte ratio (NLR), platelet-to-lymphocyte ratio (PLR), systemic immune-inflammation index (SII), C-reactive protein (CRP), lymphocyte-to-C-reactive protein ratio (LCR), and multi-inflammatory index (MII). Numerous studies have clearly shown that these novel inflammatory markers can predict the prognosis of patients with APE [7-15]. These markers can be easily calculated and readily accessed from a blood count.

The relationship between novel inflammatory markers and the clinical outcomes of thrombolytic treatment is still not well understood. This study aims to examine how these new inflammatory markers relate to thrombolytic treatments in high-risk APE patients.

## Materials and methods

Ethics approval

The study received ethical approval from the Ethics Committee of Necmettin Erbakan University Medical Faculty (Approval no. 20030, 28 June 2024). Additionally, informed consent was obtained from all subjects participating in the study. The research was conducted in accordance with international ethical guidelines, including the Declaration of Helsinki.

Subjects

Study Design

This retrospective, single-center study enrolled a total of 150 APE patients at Necmettin Erbakan University Meram Medical Faculty Hospital from June 2019 to April 2023. The diagnosis of APE was confirmed using CTPA.

Inclusion Criteria

The main inclusion criterion for the study was having APE and receiving treatment with thrombolytics.

Exclusion Criteria

Patients were excluded for the following reasons: pregnancy; acute coronary syndrome; use of anticoagulants, steroids, or immunosuppressive drugs that could affect test results; hematological diseases; acute or chronic infections; being under 18 years of age, or missing data.

A total of 69 APE patients who received thrombolytics, based on the European Society of Cardiology (ESC) guidelines for the diagnosis and management of APE, were identified from hospital records and patient files. Additionally, a control group of 69 patients without pulmonary embolism was selected from the cardiology department. This control group was matched for age and comorbidities and was selected between June 2023 and May 2024.

Methods

Blood samples were collected from patients upon their first admission for APE and from control groups, as well as before discharge for patients who received thrombolytics via the peripheral venous route. Complete blood counts were analyzed using a Siemens Advia 2120 Device (Siemens Healthcare Diagnostics, Chicago, IL, USA), while biochemical analyses were conducted with a Siemens Atellica Solution Device (Siemens Healthineers, Erlangen, Germany) employing standard biochemical techniques.

The severity of APE was classified according to the European Society of Cardiology Guidelines into high-risk, intermediate-risk, and low-risk categories. This classification was based on the presence of right ventricular (RV) dysfunction as observed on echocardiography, systolic blood pressure in the emergency department, and elevated plasma troponin levels. Patients classified as high-risk APE received thrombolytic therapy with recombinant tissue plasminogen activator.

The NLR, PLR, and SII were defined as the ratios of neutrophils to lymphocytes, platelets to lymphocytes, and the product of platelets and neutrophils divided by lymphocytes, respectively. Additionally, the MII-1 was defined as the NLR multiplied by CRP, MII-2 as the PLR multiplied by CRP, and the LCR as the ratio of lymphocytes to CRP.

Statistical analysis

The data collected from the study were transferred to a computer and analyzed using IBM SPSS Statistics for Windows, Version 27.0 (Released 2020; IBM Corp., Armonk, New York, United States). Descriptive analyses presented frequency data as numbers (n) and percentages (%), while numerical data were reported as means ± standard deviations (SD) and medians with their interquartile ranges (Q1 (first quartile) - Q3 (third quartile)). The normality of numerical data was assessed using both visual methods (histograms and Q-Q plots) and analytical methods (Kolmogorov-Smirnov test). For numerical variables that followed a normal distribution, the independent samples t-test was employed to compare two independent groups. In contrast, the Mann-Whitney U test was applied for numerical variables that did not adhere to a normal distribution. To compare two dependent groups, the paired samples t-test was used for normally distributed variables, while the Wilcoxon test was utilized for those that were not. The Pearson chi-square (χ²) test was used for comparing categorical data. The relationship between two numerical variables was investigated using Spearman correlation analysis. Correlation levels were interpreted as follows: r = 0.05-0.30 indicated a low degree of correlation, r = 0.30-0.40 indicated a low to moderate degree of correlation, r = 0.40 - 0.60 indicated a moderate degree of correlation, r = 0.60-0.70 indicated a good degree of correlation, r = 0.70-0.75 indicated a very good degree of correlation, and r = 0.75-1.00 indicated an excellent degree of correlation. A p-value <0.05 was considered statistically significant for all tests.

## Results

A total of 138 individuals participated in the study, which included both patients with pulmonary embolism and a control group without pulmonary embolism, as detailed in Table 1. Of the participants, 50% were diagnosed with pulmonary embolism. The demographic data revealed that 56.5% of the participants were female, while 43.5% were male. Additionally, 72.5% of the cases had diabetes mellitus (DM), 61.5% had hypertension (HT), and 84.7% had chronic kidney disease (CKD).

**Table 1:** Demographic and clinical characteristics of participants. CKD: chronic kidney disease; DM: diabetes mellitus; HT: hypertension.

Characteristics	Groups (patients and control)	n (%)
Pulmonary embolism	Yes	69 (50.0)
	No	69 (50.0)
Sex	Women	78 (56.5)
	Men	60 (43.5)
DM	No	100 (72.5)
	Yes	38 (27.5)
HT	No	85 (61.6)
	Yes	53 (38.4)
CKD	No	116 (84.7)
	Yes	22 (15.3)

Table 2 shows no statistically significant differences between the control and pulmonary embolism groups regarding sex, DM, HT, and CKD characteristics (p>0.05).

**Table 2:** Comparison of the control group and the pulmonary embolism group based on gender and the prevalence of chronic diseases. Pearson chi-square (χ²) was used in Table 2, and the level of statistical significance was set at p<0.05. CKD: chronic kidney disease; DM: diabetes mellitus; HT: hypertension.

Characteristics		Control n (%)	Pulmonary embolism n (%)	p-value	χ^2^
Sex	Women	35 (50.7)	43 (62.3)	0.170	1.887
	Men	34 (49.3)	26 (37.7)		
DM	No	47 (68.1)	53 (76.8)	0.253	1.307
	Yes	22 (31.9)	16 (23.2)		
HT	No	37 (53.6)	48 (69.6)	0.054	3.707
	Yes	32 (46.4)	21 (30.4)		
CKD	No	58 (84.1)	58 (85.3)	0.841	0.040
	Yes	11 (15.9)	10 (14.7)		

Table 3 shows a statistically significant increase in the levels of CRP, WBC, SII, neutrophil, NLR, and MII-1 in the pulmonary embolism group compared to the control group (p<0.001, p<0.001, p<0.001, p<0.011, p<0.013, p<0.003, p<0.001, p<0.001, and p<0.001, respectively). Conversely, there was a significant decrease in hemoglobin, MII-2, and LCR levels (p<0.011, p<0.001, and p<0.001, respectively). No statistically significant differences were observed in the other parameters (p>0.05).

**Table 3:** Comparison of pre-thrombolytic treatment values between the control group. ^1^An independent samples t-test was performed. Mean±SD is reported. ^2^The Mann-Whitney U test was conducted, and the median (Q1-Q3) was reported. The level of statistical significance was set at p<0.05. CRP: C-reactive protein; NLR: neutrophil-to-lymphocyte ratio; PLR: platelet-to-lymphocyte ratio; SII: systemic immune-inflammation index; LCR: lymphocyte-to-C reactive protein ratio; MII: multi-inflammatory index; WBC: white blood cell, L: liter; dl: deciliter; ul: microliter; mg: milligram.

Parameters	Control (n=69)	Pulmonary embolism (n=69)	t/U	p-value
CRP, mg/L	3.00 (1.38-6.53)	48.00 (20.50-103.50)	1.735	0.001^2^
WBC, 10^3^/uL	8.69±2.56	11.42±3.88	-4.872	0.001^1^
Neutrophil, 10^3^/uL	5.83±2.24	8.46±3.53	-5.222	0.001^1^
Lymphocyte, 10^3^/uL	2.07±0.92	2.21±1.14	-0.830	0.408^1^
Hemoglobin, g/dL	13.83±1.88	12.93±2.21	2.567	0.011^1^
Platelet, 10^3^/uL	230.00 (200.00-290.00)	241.00 (183.50-285.50)	2.365	0.949^2^
SII, 10^3^/uL	594.42 (413.15-993.05)	817.23 (577.61-1.440.97)	1.796	0.013^2^
NLR, uL	2.47 (1.90-3.88)	4.00 (2.41-6.44)	1.677	0.003^2^
PLR, uL	122.25 (92.01-153.56)	111.81 (82.15-170.96)	2.205	0.455^2^
MII-1, uL/mg	8.13 (2.97-21.27)	178.83 (52.60-586.39)	3.030	0.001^2^
MII-2, uL/mg	330.43 (164.34-762.35)	5.340.00 (1.772-15.366)	3.520	0.001^2^
LCR, uL/mg	0.69 (0.27-1.42)	0.04 (0.01-0.13)	3.280	0.001^2^

In Table 4, the evaluation of the pulmonary embolism group was conducted in two phases: pre- and post-thrombolytic treatment. The results indicated that post-thrombolytic treatment levels of CRP, WBC, neutrophils, lymphocytes, hemoglobin, SII, NLR, and MII-1 were significantly lower compared to pre-thrombolytic treatment levels (p<0.001, p<0.001, p<0.001, p<0.036, p<0.001, p<0.016, p<0.001, and p<0.001, respectively). Conversely, the post-thrombolytic group showed significantly higher levels of platelets, PLR, and LCR than the pre-thrombolytic group (p<0.020, p<0.018, and p<0.005, respectively). There was no statistically significant difference observed between the two groups at the MII-2 level (p>0.05).

**Table 4:** Comparison of pre-and post-thrombolytic treatment values in pulmonary embolism patients. ^1^A paired samples t-test was performed. Mean±SD is reported. ^2^The Wilcoxon test was conducted. The median (Q1-Q3) is reported. The level of statistical significance was set at p<0.05. CRP: C-reactive protein; NLR: neutrophil-to-lymphocyte ratio; PLR: platelet-to-lymphocyte ratio; SII: systemic immune-inflammation index; LCR: lymphocyte-to-C-reactive protein ratio; MII: multi-inflammatory index; WBC: white blood cell, L: liter; dl: deciliter; ul: microlitre; mg: milligram.

	Pre-thrombolytic	Post-thrombolytic	t/Z	p-value
CRP, mg/L	48.00 (20.50-103.50)	32.96 (15.00-65.00)	-5.012	0.001^2^
WBC, 10^3^/uL	10.80 (9.35-12.95)	7.70 (6.50-10.10)	-5.082	0.001^2^
Neutrophil, 10^3^/uL	8.46±3.53	5.59±2.76	7.005	0.001^1^
Lymphocyte, 10^3^/uL	2.21±1.14	1.92±0.68	2.144	0.036^1^
Hemoglobin, g/dL	13.00 (11.20-14.70)	11.40 (10.35-13.10)	-4.792	0.001^2^
Platelet, 10^3^/uL	241.00 (183.50-285.50)	271.00 (218.00-336.50)	-2.335	0.020^2^
SII, 10^3^/uL	817.23 (577.61-1.440.97)	650.53 (458.75-1.204.21)	-2.413	0.016^2^
NLR, uL	4.00 (2.41-6.44)	2.62 (1.84-4.40)	-4.003	0.001^2^
PLR, uL	111.81 (82.15-170.96)	144.37 (107.57-199.54)	-2.371	0.018^2^
MII-1, uL/mg	178.83 (52.60-586.39)	90.57 (28.06-224.21)	-4.870	0.001^2^
MII-2, uL/mg	5.340.00 (1.772.19-15.366.66)	4.581.81 (1.680-11.599)	-1.552	0.121^2^
LCR, uL	0.04 (0.01-0.13)	0.05 (0.02-0.14)	-2.801	0.005^2^

## Discussion

This study is the first to explore the relationship between novel inflammatory markers and thrombolytic treatment in patients with APE. Initially, we compared the control group with the APE group at the time of diagnosis. The results indicated that levels of CRP, WBC, neutrophils, SII, NLR, MII-1, and MII-2 were significantly higher in the APE group than in the control group. Next, we compared the APE group’s values at diagnosis with their values after thrombolytic treatment. The results showed that levels of CRP, WBC, neutrophils, lymphocytes, hemoglobin, SII, NLR, and MII-1 were significantly lower following treatment than before treatment. These findings highlight the potential role of these novel inflammatory markers in indicating the effectiveness of thrombolytic therapy in patients with APE.

Inflammatory mediators, such as CRP, interleukins (IL-6 and IL-8), and tumor necrosis factor-alpha (TNF-α), play significant roles in both arterial and venous thrombosis. Inflammation and platelet activation are crucial factors in the pathophysiology and prognosis of pulmonary embolism [16,17]. Thrombosis can worsen inflammation, while excessive inflammatory responses may trigger thrombus formation by activating platelets, leukocytes, and endothelial cells [18,19]. Effective medical treatment can break this cycle of inflammation and thrombosis, ultimately improving patient outcomes.

The CRP is an acute-phase protein that exhibits both proinflammatory and anti-inflammatory properties. It responds to cytokines such as IL-6, IL-1, and TNF [20,21]. CRP has gained recognition as a diagnostic and prognostic marker for various conditions, including autoimmune diseases, cardiovascular diseases, chronic kidney disease, cancer, and infectious diseases. As a readily measurable biochemical marker, CRP can aid in risk stratification and inform therapeutic management for patients with APE [16,22]. In line with existing literature, our study found that CRP levels were significantly elevated in the APE group compared to the control group. This elevation is likely linked to vessel damage associated with APE. Furthermore, the significant decrease in CRP levels following thrombolytic treatment indicates its effectiveness in reducing thrombus burden and the resultant damage to the vessel wall.

SII is a simple, cost-effective, and noninvasive clinical marker derived from the counts of neutrophils, platelets, and lymphocytes. SII is strongly linked to the incidence and prognosis of various cancers, and it can predict survival outcomes across different cancer types [23]. Additionally, it has been utilized as a prognostic marker in several diseases, where high SII levels correlate with poorer outcomes, increased mortality, and a heightened incidence of hypertension in stroke patients [24]. Furthermore, elevated SII levels are independently associated with a greater risk of future cardiac events, such as cardiac death, nonfatal myocardial infarction, stroke, or hospitalization for heart failure in patients with coronary artery disease [25]. Previous studies have demonstrated that SII is independently correlated with high-risk APE, emphasizing its prognostic value [7]. In our study, SII levels were significantly higher in the APE group compared to the control group, likely due to elevated serum levels of procoagulatory and proinflammatory microparticles from platelets, leukocytes, and endothelial cells present in APE. Additionally, SII levels significantly decreased following thrombolytic treatment, indicating that SII could serve as a valuable marker for assessing treatment progress.

The NLR, derived from a complete blood count with differential, is an inexpensive, easily accessible, and widely used marker of inflammation [26]. NLR has proven to be a valuable prognostic indicator in cardiovascular diseases, infections, inflammatory conditions, and various cancers [27]. Neutrophils are the first leukocytes to infiltrate damaged pulmonary tissue, and elevated neutrophil levels, along with a high NLR, have been consistently observed in patients with APE [28]. This aligns with our findings, where the APE group exhibited significantly higher NLR levels compared to the control group. Previous research suggests that a high NLR is a strong predictor of mortality in APE patients [29]. Additionally, the significant decrease in NLR following thrombolytic treatment in our study may indicate a reduction in inflammation within the pulmonary arteries, as well as a corresponding decrease in mortality risk.

The MII-1 and MII-2 are novel inflammatory markers calculated from hemogram parameters. Recent studies have established a connection between these indices and the prognosis and clinical severity of inflammation-related conditions, such as cancers, pulmonary events, and viral diseases [30,31]. Our study found that both MII-1 and MII-2 levels were significantly higher in the APE group compared to the control group. According to Boyuk, the MII may act as a robust and independent indicator of embolism severity, potentially surpassing traditional blood markers such as CRP and troponin [11,15]. 

Importantly, our results indicated a significant decrease in MII-1 levels following thrombolytic treatment, while MII-2 levels remained unchanged. This discrepancy is likely due to the different components used in their calculations: MII-1 includes the NLR, whereas MII-2 incorporates the PLR. Previous research has shown that the PLR is significantly higher in patients with low-risk APE compared to those with high-risk APE and may provide prognostic value for predicting three-month mortality, while the NLR is more indicative of in-hospital outcomes [11]. Given that all patients in our study were classified as high-risk pulmonary embolism cases and received thrombolytic treatment, with the second blood sample taken before discharge, this may explain why MII-1 showed a significant change, while MII-2 did not. APE is a major cause of cardiovascular mortality and morbidity. Timely diagnosis and prompt treatment are crucial for suspected cases [2]. New indicators of inflammation and thrombosis, such as SII, NLR, MII-1, and MII-2, may prove useful for monitoring the inflammatory response and evaluating the effectiveness of therapeutic interventions [7,8].

Limitations of study

The primary limitations of our study include its single-center design and retrospective data. Although our dataset is relatively complete, the small sample size is a significant limitation. The use of CRP instead of high-sensitivity CRP (hs-CRP) was another limitation of the study. We did not have RV function values on echocardiography, which was another limitation of our study. Consequently, although our findings may not be widely generalizable, they offer valuable insights that could inform future research aimed at yielding more reliable and conclusive results.

## Conclusions

Based on our findings, we determined that the SII, NLR, and MII-1 are strongly associated with thrombolytic treatment in patients with APE. Post-thrombolytic levels of these markers were significantly lower compared to their pre-treatment values. These markers, which can be easily calculated using complete blood count and CRP measurements, may be useful for assessing treatment effectiveness and monitoring inflammatory processes. However, prospective multicenter clinical trials are needed to validate our study's findings. In the future, the diagnosis of pulmonary embolism is expected to occur more rapidly, leading to reduced mortality due to earlier treatment. New targeted therapies may be developed that focus on inflammation and hemostasis. Additionally, the range of inflammatory markers is likely to expand, becoming increasingly crucial for both diagnosis and treatment.
